# Integrated genotype–phenotype analysis of familial adenomatous polyposis-associated hepatocellular adenomas

**DOI:** 10.1007/s00428-023-03680-w

**Published:** 2023-10-23

**Authors:** Marcell Tóth, Martina Kirchner, Thomas Longerich, Albrecht Stenzinger, Peter Schirmacher

**Affiliations:** Institute of Pathology, University Hospital Heidelberg, Im Neuenheimer Feld 224, 69120 Heidelberg, Germany

**Keywords:** Familial adenomatous polyposis, Adenomatous polyposis coli gene, Gene mutation, Liver tumor, Hepatocellular adenoma, Molecular subtype

## Abstract

Familial adenomatous polyposis (FAP) is an autosomal dominant syndrome caused by a germline mutation in the adenomatous polyposis coli (*APC*) gene, characterized by numerous colorectal adenomas. In addition, FAP patients may develop extraintestinal manifestations. Several cases of hepatocellular adenomas (HCA) detected accidentally in FAP patients have raised the so-far unsolved question of whether they represent a specific manifestation of FAP or a mere coincidence. To investigate the incidence of liver tumors in FAP patients, we analyzed our diagnostic database from 1991 to 2021. Among the 58 hepatic mass lesions identified, five HCAs occurring in three patients with FAP were identified, and comprehensive morphological, immunohistological, and molecular analysis employing targeted next-generation sequencing was conducted for characterization. The HCAs in this study showed no cytological or histological atypia. They displayed a diffuse, strong positivity for glutamine synthetase but no nuclear beta-catenin immunostaining. In two patients, the adenomas showed moderate immunoreactivity against serum amyloid A. Consistent with the diagnosis of FAP, molecular profiling revealed a pathogenic germline mutation of the APC gene in all analyzed adenomas as well as deleterious somatic second hits. All somatic mutations were localized between codons 1345 and 1577. No mutations were found in the catenin beta 1 gene. HCA in FAP patients can be a specific, although rare, neoplastic manifestation of this inborn disease and represents a distinct subgroup of HCAs. These benign tumors represent an important differential diagnosis for hepatic metastases in FAP patients and require adequate clinical and molecular (diagnostic) assessments for optimal patient guidance.

**Supplementary Information:**

The online version contains supplementary material available at 10.1007/s00428-023-03680-w.

## Introduction

Hepatocellular adenomas (HCAs) are benign liver neoplasms which predominantly (ca. 85% of the cases) occur in women during their reproductive period [1]. The major risk factors are the intake of oral contraceptives, anabolic steroids, and androgens [2–4]. HCAs may be solitary or multiple, with a condition of at least 10 HCAs called adenomatosis [5]. Different subtypes of HCA with distinct biological behavior, therapeutic need, and prognosis can be distinguished histologically, immunohistologically, and by molecular analyses [1, 6]. Hepatic nuclear factor 1 alpha (*HNF1A*)-inactivated HCAs account for 30–35% of all HCAs and are typically characterized by diffuse steatosis and loss of liver fatty-acid binding protein (LFABP) expression. Inflammatory HCAs display inflammatory foci, sinusoidal dilatation, ductular reaction, and diffuse positivity for C-reactive protein (CRP) and serum amyloid A (SAA). They represent 35–40% of HCAs. The third major subtype, with 20–25%, is beta-catenin-activated HCA. These tumors often show pseudoglandular growth patterns and cytological atypia, feature activating mutations of the catenin beta 1 (*CTNNB1*) gene, and depending on the mutation type, different patterns of overexpression of glutamine synthetase (GS) [7, 8]. About 10% of inflammatory HCAs may also exhibit a mutation in the *CTNNB1* gene and are referred to as beta-catenin-activated inflammatory HCAs [1]. Recently, a new subtype of HCA was described, which represents ca. 4% of HCAs, which are associated with obesity and bleeding risk, are defined by the activation of the sonic hedgehog signaling pathway, and are due to the fusion of the inhibin subunit beta E and glioma-associated oncogene 1 (*GLI1*) genes [9]. About 5% of HCAs cannot be subtyped according to morphomolecular characteristics, so far [10].

Familial adenomatous polyposis (FAP) is an autosomal dominant syndrome caused by a germline mutation in the adenomatous polyposis coli (*APC*) gene [11]. The disease is characterized by numerous (> 100) colorectal adenomas, developing during childhood and adolescence [12–14]. Few of these polyps progress through the adenoma-adenocarcinoma sequence, resulting in a cumulative lifetime risk of colorectal adenocarcinoma of almost 100% [15].

In addition, extraintestinal manifestations may occur in FAP patients [16]. Approximately 10% of the patients develop desmoid tumors [17]. Benign tumors, such as osteomas or odontomas, are also commonly observed [14]. Hepatoblastomas can also be associated with FAP [18, 19]. Infrequently, HCAs were also reported in FAP patients. So far, nine such cases of HCAs in FAP patients have been described in the scientific literature, and it has remained unclear whether they represent a specific manifestation of FAP or a mere coincidence (Table 1) [1, 20–27]. Typically, these tumors are asymptomatic, detected incidentally, and diagnosed in staging or follow-up diagnostics due to colorectal cancer. Here, we performed comprehensive morphological, immunohistological, and molecular analyses which describe a peculiar subgroup of HCA as a rare, specific manifestation of FAP.

**Table 1:** Reported cases of FAP-associated hepatocellular adenomas. The table contains clinical, pathological, and molecular pathological information about the previously reported, and in this study discussed HCA cases in FAP patients

Age/sex	Site	Multiplicity	Subtype	APC germline mutation (codon)	APC somatic mutation (codon)	Reference
2F	Right lobe	Solitary	-	1451	-	Bala et al. 1997 [20]
20/F	Left lobe	Multiple	-	-	-	Nakao et al. 2000 [21]
37/F	Right lobe	Solitary	H-HCA	1062	-	Jeannot et al. 2006 [23]
27/M	Left lobe	Solitary	-	-	-	Okamura et al. 2009 [24]
25/M	Left lobe	Solitary	H-HCA	-	-	Toiyama et al. 2011 [25]
29/F	Right lobe	Solitary	HCA-NOS	499	-	Inaba et al. 2012 [26]
M	S5/6	Solitary	I-HCA	-	-	Bioulac-Sage et al. 2013 [1]
37/F	-	> 10	B-HCA	-	-	Crimi et al. 2018 [27]
22/F	S4b	Solitary	I-HCA	1156	1517	Blaker et al. 2004/patient 2 [22]
57/M	Right lobe (S6)	Solitary	B-HCA/I-HCA	1465	1345	Patient 3
25/M	S4b	Multiple	HCA-NOS	1306	1544	Patient 1/sample 2/HCA 1
25/M	S3	Multiple	HCA-NOS	1306	1566	Patient 1/sample 3/HCA 2
25/M	S7	Multiple	HCA-NOS	1306	1577	Patient 1/sample 4/HCA 3

*F*, female; *M*, male; *H-HCA*, HNF1A-inactivated hepatocellular adenoma; *B-HCA*, beta-catenin-activated hepatocellular adenoma; *I-HCA*, inflammatory hepatocellular adenoma; *HCA-NOS*, hepatocellular adenoma not otherwise specified

## Methods

### Patient and samples

To investigate the incidence of liver tumors in patients with FAP, we analyzed our database at the Institute of Pathology from 1991 to 2021 using the following keywords: familial adenomatous polyposis, FAP, liver, hepatocellular, HCA, and HCC. In this time period, material from 1454 FAP patients was sent to the Institute of Pathology of Heidelberg University Hospital. Of these, 58 samples represented a hepatic mass, of which 46 were found to be metastases of a colorectal-type adenocarcinoma, five were infiltrates of a pancreatobiliary adenocarcinoma, and one patient had acute myeloid leukemia. Furthermore, one patient had a biliary microhamartoma (von Meyenburg complex), and two patients had focal nodular hyperplasia (FNH); the latter were excluded from further analysis as FNHs are not considered as true neoplasms. Three additional patients (patients 1, 2, and 3) presented with liver tumors that were histologically identified as HCAs. Formalin-fixed and paraffin-embedded (FFPE) material of three HCAs, the primary colorectal adenocarcinoma and a tumor-free lymph node of patient 1, as well as FFPE material of the HCAs of patients 2 and 3, were analyzed via immunohistochemistry and targeted next-generation sequencing. The second patient has already been reported, but results from comprehensive molecular profiling were lacking [22].

### Immunohistochemistry

Three-micrometer-thick sections were cut from paraffin blocks containing tumor tissue and surrounding normal liver tissue using a microtome. After deparaffinization and rehydration, the samples were pre-treated with Cell Conditioning Solution (Ultra CC1; Ventana, Oro Valley, USA) for 32–48 min (SAA, LFABP, and GS: 32 min; beta-catenin: 40 min; and CRP: 48 min). Immunohistochemical stainings were carried out using an automated slide stainer (BenchMark Ultra system; Roche, Basel, Switzerland) using the following dilutions: SAA—1:200; LFABP—1:1000; and GS and beta-catenin—ready-to-use. The list of antibodies used in the study is provided in Supplementary Table 1.

### Hybrid capture-based panel sequencing

Patient and sample characteristics are summarized in Supplementary Table 2. DNA was extracted using a Maxwell 16 Research System (Promega, Madison, USA), followed by quantification using the QuBit 2.0 DNA High Sensitivity Kit (Thermo Fisher Scientific, Waltham, USA). Library preparation for the capture-based TruSight Oncology 500 (TSO500) panel (Illumina, San Diego, USA) was performed as previously described [28]. The panel covers all exonic regions of more than 500 genes including *APC* and *CTNNB1*. DNA integrity was assessed using the Genomic DNA ScreenTape Analysis on a 4150 TapeStation System (Agilent, Santa Clara, USA). To fragment DNA to a length of 90–250 bp, 80 ng of DNA was sheared for 50–78 s using an ME220 Focused-Ultrasonicator (Covaris, Woburn, USA). Following the target capture and purification steps, enriched libraries were amplified by 15 cycles of PCR and subsequently quality controlled using the KAPA SYBR Library Quantification Kit (Thermo Fisher Scientific) on a StepOnePlus qPCR system (Thermo Fisher Scientific). Libraries were sequenced on a NextSeq 500 instrument (Illumina) to a mean coverage of × 1096 using a high-output cartridge and v2 chemistry. All assays were performed according to the manufacturer’s protocols.

Processing of raw sequencing data and variant calling was carried out using the TSO500 Local App (version 1.3.0.39). The called variants were verified by visual inspection in the Integrative Genomics Viewer [29]. Only variants with an allele frequency above 2% and a minimum coverage of greater than × 100 were considered [30].

## Results

Patient 1—a 25-year-old male—had 16 intrahepatic nodules, ranging from 0.2 to 2.5 cm in diameter. He was diagnosed with colorectal carcinoma half a year prior to the identification of the liver nodules, which were found during follow-up and were suspected radiologically to be metastases of the colorectal carcinoma. For patient 1, no metabolic syndrome or anabolic steroid abuse was reported. The patient had the following medications at the time of the HCA diagnosis: pantoprazole, Imodium, and metamizole. Patient 2 (22-year-old female) and patient 3 (57-year-old male) presented with a solitary 5.5 cm and 4 cm hepatic mass, respectively. Both of the patients underwent a restorative proctocolectomy before the development of colorectal carcinoma. Patient 2 was assumed to have a benign hepatic lesion, which was verified to be a HCA intraoperatively during the proctocolectomy. The HCA of patient 3 was first identified at the age of 42 (15 years after the proctocolectomy) and resected at the age of 57. There was no available data about oral contraceptive use or serum CRP levels for patient 2. Patient 3 had no anabolic steroid abuse. He only had substitution therapy with L-thyroxin after a thyroidectomy. His serum CRP level was not elevated.

**Fig. 1:**
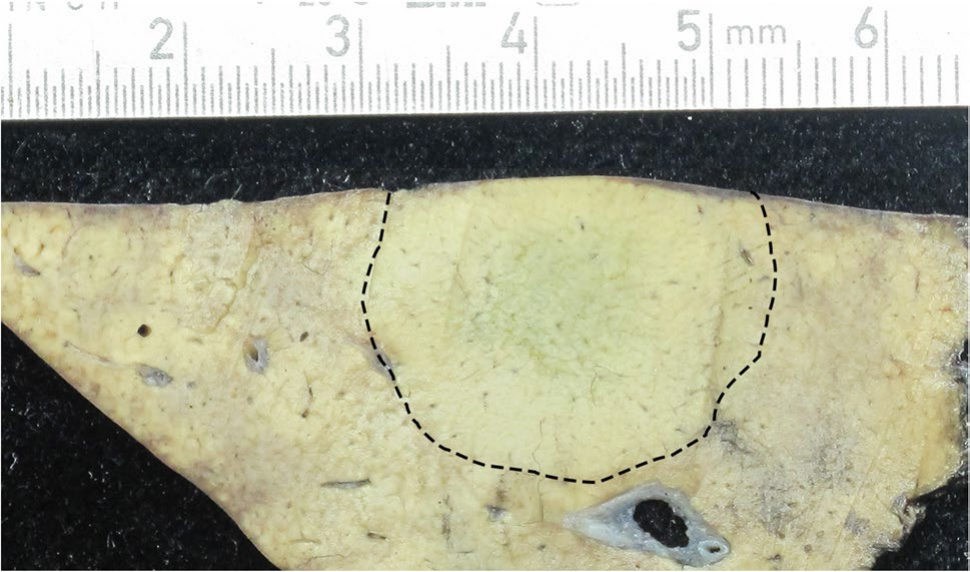
Macroscopic findings of FAP-associated HCAs. Macroscopic examination of the surgical specimen (segment 3) from patient 1 revealed a subcapsular, well-circumscribed, 2.5 cm lesion with a tan-colored surface without hemorrhage or necrosis. Dashed line: border between HCA and surrounding normal liver tissue

On gross examination, the lesions were soft, circumscribed, and tan-colored (Fig. 1). Microscopically, the tumors were not encapsulated and displayed a trabecular growth pattern consisting of one- to two-cell-wide hepatocellular plates. The detailed pathomorphological findings are listed in Supplementary Table 3. According to the histological criteria, all lesions were classified as HCAs (accordingly, adenomatosis in patient 1). The tumor cells showed no significant cytological atypia. HCAs of patient 1 displayed micro- and macrovesicular steatosis in approximately 60% of the tumor cells, while HCAs of the other two patients showed significant intratumoral inflammation and sinusoidal dilatation.

**Fig. 2:**
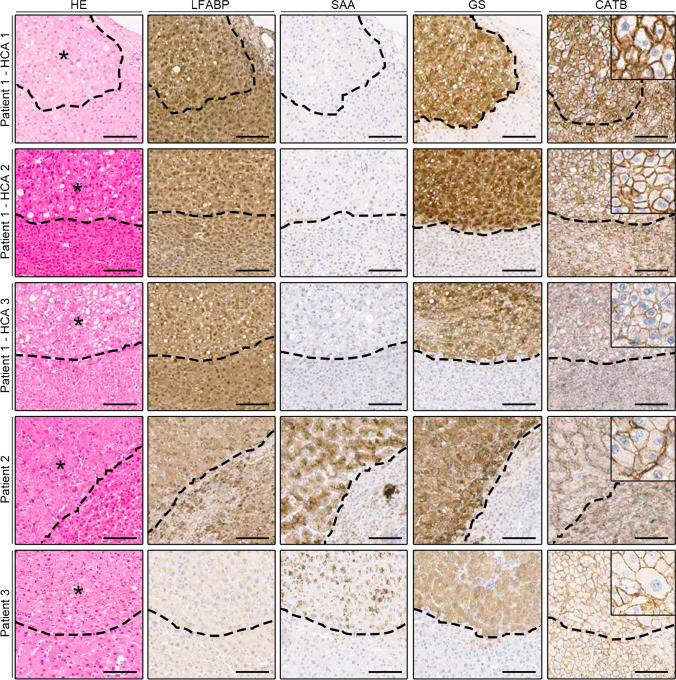
Immunohistochemical analysis of FAP-associated HCAs. Representative hematoxylin–eosin and immunohistochemical stains (LFABP, SAA, GS, and beta-catenin (CATB)) in five analyzed HCAs from three patients. Asterisk: HCA tissue. Note that the immunoreactivity in case 3 is generally weaker. Dashed line: border between HCA and surrounding normal liver tissue. Magnification: × 40; magnification of index pictures: × 100. Bars: 50 µm

The immunohistological stains showed a diffuse, strong positivity of GS in all tumors (Fig. 2). Beta-catenin immunostaining displayed diffuse membranous but no nuclear positivity, and there was no loss of LFABP expression in the lesions compared to the surrounding liver parenchyma. HCAs in the second and third cases showed specific immunoreactivity for SAA, while tumors in the first patient were negative for SAA.

**Fig. 3:**
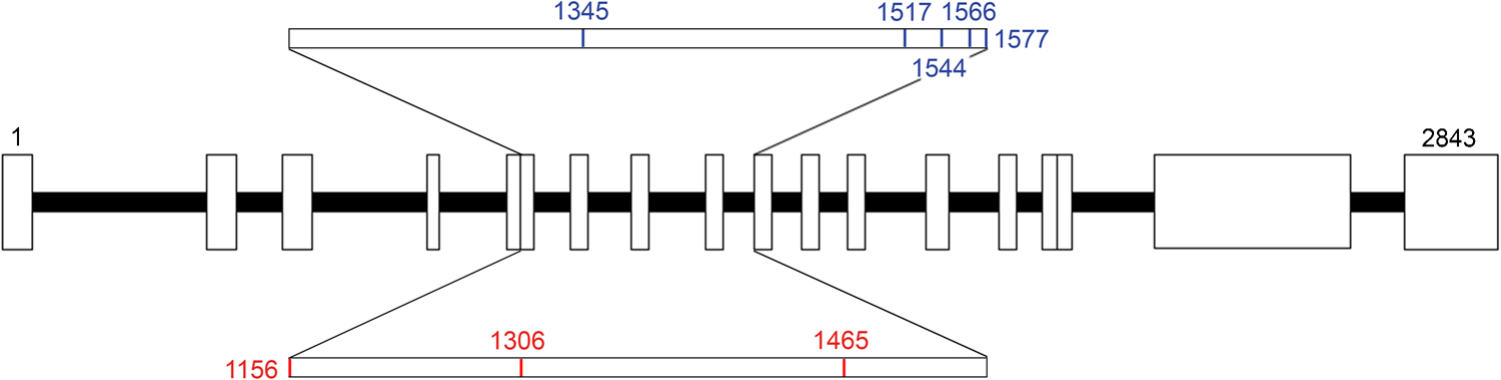
Distribution of germline and somatic mutations in the APC-gene. Scheme of the APC gene showing the detected germline and somatic mutations in the HCAs. Mutations accumulated between codons 1156 and 1577 (zoomed-in area). Red color, germline mutations; blue color, somatic mutations

Molecular profiling of three separate liver nodules, the primary adenocarcinoma of the rectum, and a tumor-free lymph node from patient 1 identified a pathogenic germline mutation in the *APC* gene and different deleterious somatic mutations in the colorectal adenocarcinoma and the liver nodules (Fig. 3). Molecular characterization of the HCAs from patients 2 and 3 also showed loss-of-function mutations in the *APC* gene. Furthermore, a *JAK3* variant of unknown significance was discovered in the HCA of patient 3. No genetic alterations were found in the *HNF1A* and *GLI1* genes or the analyzed JAK/STAT pathway members (*JAK1*, *GNAS*, *ROS1*, and *STAT3*) in any of the HCAs in this cohort. All the analyzed adenomas showed only variants of unknown significance in addition to the inactivating mutations of the *APC* genes regardless of the number of HCAs in the patients. An overview of the *APC* mutations and additional mutations detected in the study cohort is presented in Table 2 and Supplementary Table 4. All tumors were microsatellite-stable (MSS).

**Table 2:** Overview of APC mutations in the presented cases. The table describes the germline and somatic mutations in the colorectal carcinoma (sample 1), HCAs (samples 2–4), and tumor-free lymph nodes (sample 5) of the first patient and in the HCAs of the second and third patient

Patient/sample	Gene	Exon	Mutation (c.DNA)	Mutation (protein)	Allele frequency (%)	Variant
Patient 1/sample 1	APC (NM_000038.6)	7	c.694C > T	p.Arg232*	19.2	Lof
		16	c.3916_3917insGGTATT	p.Glu1306fs*3	35.2	Lof
Patient 1/sample 2	APC (NM_000038.6)	16	c.4630G > T	p.Glu1544*	3.8	Lof
		16	c.3916_3917insGGTATT	p.Glu1306fs*3	43.3	Lof
Patient 1/sample 3	APC (NM_000038.6)	16	c.4666dupA	p.Thr1566fs*3	30	Lof
		16	c.3916_3917insGGTATT	p.Glu1306fs*3	47.8	Lof
Patient 1/sample 4	APC (NM_000038.6)	16	c.4729G > T	p.Glu1577*	16.3	Lof
		16	c.3916_3917insGGTATT	p.Glu1306fs*3	44	Lof
Patient 1/sample 5	APC (NM_000038.6)	16	c.3916_3917insGGTATT	p.Glu1306fs*3	44	Lof
Patient 2	APC (NM_000038.6)	16	c.4544_4547dup	p.Gln1517fs*17	11.6	Lof
		16	c.3467_3470del	p.Glu1156fs*8	41.4	Lof
		16	c.5540C > T	p.Thr1847Met	40.2	VUS
Patient 3	APC (NM_000038.6)	16	c.4033G > T	p.Glu1345*	11	Lof
		16	c.4391_4392del	p.Ser1465fs*3	43.5	Lof

*Lof*, loss of function; *VUS*, variant of unknown significance

Next, we performed a comprehensive analysis of the cases reported in the literature in order to compare these data with our results (see Table 1). The previously described FAP-associated HCAs occurred both in female and male patients (ratio 2:1). The youngest patient was two, and the oldest was 37 years of age at the time of diagnosis. The size of the HCAs ranged from 2.8 to 10 cm. Most of the HCAs were solitary, but in two cases, multiple tumors were described [21, 27].

Histologically, the HCAs were described as well-differentiated nodules with a trabecular growth pattern lacking any histological or cytological atypia. Immunohistological analysis was carried out only in three cases; two HCAs showed diffuse and strong GS positivity [1, 27]. In the third case, the GS stain was negative, and the HCA was classified as *HNF1A*-inactivated [23].

A molecular analysis was performed only in three cases so far. Bala et al. found in the reported HCA a germline *APC* mutation at codon 1451 and deletion of the second allele [20]. In two further cases, a germline mutation of *APC* was found (codons 1062 and 499), without a second hit [23, 26]. However, it was not outlined whether the whole *APC* gene was covered in the analysis. In addition to the germline *APC* mutation, Jeannot et al. also found a somatic mutation of *HNF1A* and classified the tumor as *HNF1A*-inactivated HCA. According to the patient history, she took oral contraception for 5 years [23]. This case may have represented a sporadic HCA occurring in a FAP patient without a pathogenetic link.

## Discussion

Few HCAs have been reported in FAP, and due to their rarity, it has been an open question whether they may represent a specific manifestation of FAP or a mere coincidence [1, 20–27]. In our study, all of our HCAs represent a clonally propagated lesion with respective genetic alterations in the *APC* gene and thus a specific manifestation of FAP—irrespective of coexisting known risk factors for HCA—since we identified mutational inactivation of the second allele in all FAP-HCAs analyzed. In contrast, the two reconfirmed FNHs found in our FAP cohort are most likely co-occurrences (though not analyzed in our study). In this context, it remains an open question why HCA is nevertheless infrequent in FAP but shows multiple HCAs in very few cases (one of our cases and two reported in the literature). There may be some HCA underreporting due to their benign nature, as they may only be detected and diagnosed in cases of manifest colorectal cancer. The spectrum of *APC* mutations being able to induce neoplastic development of hepatocytes may be limited, and also, the lower cell turnover rate of hepatocytes compared to colonic epithelium may further limit random mutational inactivation of the second allele. The occurrence of multiple HCAs in FAP may also depend on the type of germline mutation.

Inactivating mutations of the *APC* gene are distributed throughout the whole coding sequence [31]. However, a mutation cluster region for somatic mutations is located approximately between codons 1250 and 1450 for colorectal tumors and between codons 1400 and 1580 for upper gastrointestinal tumors [32]. The location of the mutations correlates to the severity of the disease in terms of colonic adenoma formation [33]. Moreover, a genotype–phenotype correlation of *APC* gene mutations has also been described regarding extraintestinal manifestations. Mutations located between codons 1445 and 1580 are associated with an elevated risk and severe manifestation of desmoid tumors, while mutations between 457 and 1309 increase the risk of hepatoblastomas [16, 34]. Mutations occurring after codon 1444 correlate with a higher risk of osteomas [35]. In our study, the analysis of the *APC* gene revealed that the mutations that occurred in the HCAs (both somatic and germline) were located between codons 1156 and 1577 (Fig. 3). All somatic mutations localized between codons 1306 and 1577, and four out of the five somatic mutations were in the previously described mutation cluster region [32].

The HCAs seen in our collective share some morphological characteristics: our cases do not show significant cytological or histological atypia. All cases we have included show strong and diffuse activation of GS but lack nuclear accumulation of beta-catenin and *CTNNB1* mutations. Among the previously described HCAs, immunohistochemistry was only performed in three cases, from which in two cases a strong and diffuse GS staining was revealed [1, 27]. Cytological or histological atypia was not reported in any of the previously published cases.

In this study, we evaluated the cases of two male (25 and 57 years old) and one female (22 years old) patients. The largest nodule in the first case with adenomatosis was 2.5 cm, while the adenomas in the other two cases were 5.5 cm and 4 cm in diameter. In the previously described HCA cases, the patients were between 2 and 37 years old, and the HCAs measured between 2.8 and 10 cm at the time of detection. The age of the patients and the size at detection do not differ from the established HCA subtypes. However, a gender disparity in FAP-HCAs is not detectable so far (six female and five male patients).

An important question is how FAP-HCA relates to other subtypes of HCA. At first glance, the subtype appears to be most closely related to beta-catenin-activated HCA, as the *APC* gene, which is part of the destruction complex in the canonical Wnt-signaling pathway and a negative regulator of beta-catenin, shows deleterious variants in both alleles and as all FAP-HCAs show strong and homogenous upregulation of GS, a Wnt-signaling target gene [36, 37]. On the other hand, there are significant differences questioning whether FAP-HCA should be added to the subgroup of beta-catenin-activated HCA with conventional exon 3 mutations of the *CTNNB1* gene and strong and homogenous GS overexpression. First of all, we detected no activating mutation of *CTNNB1* in FAP-HCA, and—consistent with this finding—there is no nuclear beta-catenin accumulation in any of the HCA cells, as it is otherwise consistently found in HCA with homogenous, strong GS overexpression. Furthermore, all FAP-HCAs investigated here lacked significant cellular or architectural atypia, which is frequently seen in beta-catenin-activated HCA. Accordingly, the FAP-HCAs in our collective lack signs for an increased risk of malignant transformation, which in contrast is the case for beta-catenin-activated HCA, and there is only a single case report on a FAP-HCA with malignant transformation in the literature [25]. Loss-of-function mutations in *APC* are also rarely found in HCC (less than 3%) [38]. Accordingly, only ten cases of FAP-associated HCCs have been described in the literature so far. Among these FAP-associated HCCs, molecular analysis was performed only in three cases, and only in a single case was a somatic mutation in the *APC* gene found [39]. In none of these cases, an HCC precursor lesion or adenoma was described. Interestingly, the age of the patients at the time of HCA diagnosis (reviewed in this article) and the age of the patients at the time of HCC diagnosis did not differ significantly. Mutational inactivation of the *APC* gene alone was also found to be insufficient to promote hepatocarcinogenesis in a *sgApc* mouse model, and additional genetic events were needed to induce HCC formation [38].

Overall, these data substantiate the claim that FAP-HCA forms a peculiar subgroup of HCA. Interestingly, like in beta-catenin-activated HCA, presumed alteration of the Wnt-signaling pathway in FAP-HCA may also co-occur with the inflammatory phenotype of HCA. In addition, it also shows that in some rare cases of HCA (i.e., FAP-HCA), strong overexpression of GS alone does not allow to ascribe a HCA to the subtype of beta-catenin-activated HCA and may not necessarily demonstrate an increased risk of malignant transformation.

Which are the clinical consequences? First of all, the vast majority of FAP-HCAs will be found accidentally under staging/restaging conditions of colorectal cancer, with hepatic metastasis being the clinical suspicion or at least differential diagnosis due to probability. This lends further evidence to the need for a biopsy of suspicious hepatic lesions in the respective FAP patients. If adenoma would be detected by biopsy and its FAP-related nature can be clarified by *APC*-gene sequencing, according to current knowledge, resection criteria may not adhere to criteria for beta-catenin-activated HCA (resection at any size) but to general HCA criteria (resection > 5 cm), and even a “watch and wait” strategy may be considered [40].

Taken together, our analyses show that hepatocellular adenoma in FAP patients can be a specific, although rare, neoplastic manifestation of this inborn disease and represents a distinct subgroup of HCA. FAP-HCA is an important differential diagnosis for hepatic metastases in these patients and requires adequate clinical and molecular (diagnostic) assessments for optimal patient guidance.

### Supplementary Information

Below is the link to the electronic supplementary material.

Supplementary file1 (DOCX 36 KB)

## Abbreviations

HCA: Hepatocellular adenoma
HNF1A: Hepatic nuclear factor 1 alpha
LFABP: Liver fatty-acid binding protein
CRP: C-reactive protein
SAA: Serum amyloid A
CTNNB1: Catenin beta 1
GLI1: Glioma-associated oncogene 1
GS: Glutamine synthetase
FAP: Familial adenomatous polyposis
APC: Adenomatous polyposis coli
FNH: Focal nodular hyperplasia
FFPE: Formalin-fixed and paraffin-embedded
TSO500: TruSight Oncology 500 panel
CATB: Beta-catenin

## References

[1] Bioulac-Sage P, Sempoux C, Possenti L, Frulio N, Laumonier H, Laurent C, Chiche L, Frederic Blanc J, Saric J, Trillaud H, Le Bail B, Balabaud C (2013). Pathological diagnosis of hepatocellular cellular adenoma according to the clinical context Int. J Hepatol.

[2] Edmondson HA, Henderson B, Benton B (1976). Liver-cell adenomas associated with use of oral contraceptives. N Engl J Med.

[3] Velazquez I, Alter BP (2004). Androgens and liver tumors: Fanconi’s anemia and non-Fanconi’s conditions. Am J Hematol.

[4] Chang CY, Hernandez-Prera JC, Roayaie S, Schwartz M, Thung SN (2013). Changing epidemiology of hepatocellular adenoma in the United States: review of the literature Int. J Hepatol.

[5] Greaves WO, Bhattacharya B (2008). Hepatic Adenomatosis Arch Pathol Lab Med.

[6] Bioulac-Sage P, Rebouissou S, Thomas C, Blanc JF, Saric J, Sa Cunha A, Rullier A, Cubel G, Couchy G, Imbeaud S, Balabaud C, Zucman-Rossi J (2007). Hepatocellular adenoma subtype classification using molecular markers and immunohistochemistry. Hepatology.

[7] Hale G, Liu X, Hu J, Xu Z, Che L, Solomon D, Tsokos C, Shafizadeh N, Chen X, Gill R, Kakar S (2016). Correlation of exon 3 beta-catenin mutations with glutamine synthetase staining patterns in hepatocellular adenoma and hepatocellular carcinoma. Mod Pathol.

[8] Rebouissou S, Franconi A, Calderaro J, Letouze E, Imbeaud S, Pilati C, Nault JC, Couchy G, Laurent A, Balabaud C, Bioulac-Sage P, Zucman-Rossi J (2016). Genotype-phenotype correlation of CTNNB1 mutations reveals different ss-catenin activity associated with liver tumor progression. Hepatology.

[9] Nault JC, Couchy G, Balabaud C, Morcrette G, Caruso S, Blanc JF, Bacq Y, Calderaro J, Paradis V, Ramos J, Scoazec JY, Gnemmi V, Sturm N, Guettier C, Fabre M, Savier E, Chiche L, Labrune P, Selves J, Wendum D, Pilati C, Laurent A, De Muret A, Le Bail B, Rebouissou S, Imbeaud S, Investigators G, Bioulac-Sage P, Letouze E, Zucman-Rossi J (2017). Molecular classification of hepatocellular adenoma associates with risk factors, bleeding, and malignant transformation. Gastroenterology.

[10] Bioulac-Sage P, Taouji S, Possenti L, Balabaud C (2012). Hepatocellular adenoma subtypes: the impact of overweight and obesity. Liver Int.

[11] Nishisho I, Nakamura Y, Miyoshi Y, Miki Y, Ando H, Horii A, Koyama K, Utsunomiya J, Baba S, Hedge P (1991). Mutations of chromosome 5q21 genes in FAP and colorectal cancer patients. Science.

[12] Haggitt RC, Reid BJ (1986). Hereditary gastrointestinal polyposis syndromes. Am J Surg Pathol.

[13] Rustgi AK (1994). Hereditary gastrointestinal polyposis and nonpolyposis syndromes. N Engl J Med.

[14] Bronner MP (2003). Gastrointestinal inherited polyposis syndromes. Mod Pathol.

[15] Vasen HF, Moslein G, Alonso A, Aretz S, Bernstein I, Bertario L, Blanco I, Bulow S, Burn J, Capella G, Colas C, Engel C, Frayling I, Friedl W, Hes FJ, Hodgson S, Jarvinen H, Mecklin JP, Moller P, Myrhoi T, Nagengast FM, Parc Y, Phillips R, Clark SK, de Leon MP, Renkonen-Sinisalo L, Sampson JR, Stormorken A, Tejpar S, Thomas HJ, Wijnen J (2008). Guidelines for the clinical management of familial adenomatous polyposis (FAP). Gut.

[16] Galiatsatos P, Foulkes WD (2006). Familial adenomatous polyposis. Am J Gastroenterol.

[17] Miyaki M, Konishi M, Kikuchi-Yanoshita R, Enomoto M, Tanaka K, Takahashi H, Muraoka M, Mori T, Konishi F, Iwama T (1993). Coexistence of somatic and germ-line mutations of APC gene in desmoid tumors from patients with familial adenomatous polyposis. Cancer Res.

[18] Garber JE, Li FP, Kingston JE, Krush AJ, Strong LC, Finegold MJ, Bertario L, Bulow S, Filippone A, Gedde-Dahl T (1988). Hepatoblastoma and familial adenomatous polyposis. J Natl Cancer Inst.

[19] Giardiello FM, Offerhaus GJ, Krush AJ, Booker SV, Tersmette AC, Mulder JW, Kelley CN, Hamilton SR (1991). Risk of hepatoblastoma in familial adenomatous polyposis. J Pediatr.

[20] Bala S, Wunsch PH, Ballhausen WG (1997). Childhood hepatocellular adenoma in familial adenomatous polyposis: mutations in adenomatous polyposis coli gene and p53. Gastroenterology.

[21] Nakao A, Sakagami K, Nakata Y, Komazawa K, Amimoto T, Nakashima K, Isozaki H, Takakura N, Tanaka N (2000). Multiple hepatic adenomas caused by long-term administration of androgenic steroids for aplastic anemia in association with familial adenomatous polyposis. J Gastroenterol.

[22] Blaker H, Sutter C, Kadmon M, Otto HF, Von Knebel-Doeberitz M, Gebert J, Helmke BM (2004). Analysis of somatic APC mutations in rare extracolonic tumors of patients with familial adenomatous polyposis coli. Genes Chromosomes Cancer.

[23] Jeannot E, Wendum D, Paye F, Mourra N, de Toma C, Flejou JF, Zucman-Rossi J (2006). Hepatocellular adenoma displaying a HNF1alpha inactivation in a patient with familial adenomatous polyposis coli. J Hepatol.

[24] Okamura Y, Maeda A, Matsunaga K, Kanemoto H, Furukawa H, Sasaki K, Yamaguchi S, Uesaka K (2009). Hepatocellular adenoma in a male with familial adenomatous polyposis coli. J Hepatobiliary Pancreat Surg.

[25] Toiyama Y, Inoue Y, Yasuda H, Yoshiyama S, Araki T, Miki C, Kusunoki M (2011). Hepatocellular adenoma containing hepatocellular carcinoma in a male patient with familial adenomatous polyposis coli: report of a case. Surg Today.

[26] Inaba K, Sakaguchi T, Kurachi K, Mori H, Tao H, Nakamura T, Takehara Y, Baba S, Maekawa M, Sugimura H, Konno H (2012). Hepatocellular adenoma associated with familial adenomatous polyposis coli World. J Hepatol.

[27] Crimi F, Guido M, Pomerri F (2018). Hepatobiliary and pancreatic: hepatic nodules in a patient with familial adenomatous polyposis and colorectal adenocarcinoma. J Gastroenterol Hepatol.

[28] Kazdal D, Endris V, Allgauer M, Kriegsmann M, Leichsenring J, Volckmar AL, Harms A, Kirchner M, Kriegsmann K, Neumann O, Brandt R, Talla SB, Rempel E, Ploeger C, von Winterfeld M, Christopoulos P, Merino DM, Stewart M, Allen J, Bischoff H, Meister M, Muley T, Herth F, Penzel R, Warth A, Winter H, Frohling S, Peters S, Swanton C, Thomas M, Schirmacher P, Budczies J, Stenzinger A (2019). Spatial and temporal heterogeneity of panel-based tumor mutational burden in pulmonary adenocarcinoma: separating biology from technical artifacts. J Thorac Oncol.

[29] Robinson JT, Thorvaldsdottir H, Winckler W, Guttman M, Lander ES, Getz G, Mesirov JP (2011). Integrative genomics viewer. Nat Biotechnol.

[30] Ronellenfitsch MW, Harter PN, Kirchner M, Heining C, Hutter B, Gieldon L, Schittenhelm J, Schuhmann MU, Tatagiba M, Marquardt G, Wagner M, Endris V, Brandts CH, Mautner VF, Schrock E, Weichert W, Brors B, von Deimling A, Mittelbronn M, Steinbach JP, Reuss DE, Glimm H, Stenzinger A, Frohling S (2020). Targetable ERBB2 mutations identified in neurofibroma/schwannoma hybrid nerve sheath tumors. J Clin Invest.

[31] Nagase H, Nakamura Y (1993). Mutations of the APC (adenomatous polyposis coli) gene. Hum Mutat.

[32] Groves C, Lamlum H, Crabtree M, Williamson J, Taylor C, Bass S, Cuthbert-Heavens D, Hodgson S, Phillips R, Tomlinson I (2002). Mutation cluster region, association between germline and somatic mutations and genotype-phenotype correlation in upper gastrointestinal familial adenomatous polyposis. Am J Pathol.

[33] Nieuwenhuis MH, Vasen HF (2007). Correlations between mutation site in APC and phenotype of familial adenomatous polyposis (FAP): a review of the literature. Crit Rev Oncol Hematol.

[34] Gebert JF, Dupon C, Kadmon M, Hahn M, Herfarth C, Doeberitz MV, Schackert HK (1999). Combined molecular and clinical approaches for the identification of families with familial adenomatous polyposis coli. Ann Surg.

[35] Gruner BA, DeNapoli TS, Andrews W, Tomlinson G, Bowman L, Weitman SD (1998). Hepatocellular carcinoma in children associated with Gardner syndrome or familial adenomatous polyposis. J Pediatr Hematol Oncol.

[36] Cadoret A, Ovejero C, Terris B, Souil E, Levy L, Lamers WH, Kitajewski J, Kahn A, Perret C (2002). New targets of beta-catenin signaling in the liver are involved in the glutamine metabolism. Oncogene.

[37] Zhan T, Rindtorff N, Boutros M (2017). Wnt signaling in cancer. Oncogene.

[38] Zhang Y, Liang B, Song X, Wang H, Evert M, Zhou Y, Calvisi DF, Tang L, Chen X (2021). Loss of Apc cooperates with activated oncogenes to induce liver tumor formation in mice. Am J Pathol.

[39] Li M, Gerber DA, Koruda M, O'Neil BH (2012). Hepatocelluar carcinoma associated with attenuated familial adenomatous polyposis: a case report and review of the literature. Clin Colorectal Cancer.

[40] European Association for the Study of the L (2016). EASL clinical practice guidelines on the management of benign liver tumours. J Hepatol.

