# Supplementary material for: Integrated genotype–phenotype analysis of familial adenomatous polyposis-associated hepatocellular adenomas
Source: Virchows Arch. 2023 Oct 23;484(4):587–95. doi: 10.1007/s00428-023-03680-w (PMC11062996; doi:10.1007/s00428-023-03680-w)
Supplement: Supplementary file 1 — Supplementary file1 (DOCX 36 KB) [file 428_2023_3680_MOESM1_ESM.docx]

**Integrated genotype-phenotype analysis of familial adenomatous polyposis-associated hepatocellular adenomas**

Marcell Tóth^1^, Martina Kirchner^1^, Thomas Longerich^1^, Albrecht Stenzinger^1^, Peter Schirmacher^1^

^1^Institute of Pathology, University Hospital Heidelberg, Im Neuenheimer Feld 224, 69120 Heidelberg, Germany

**Corresponding author:** Marcell Tóth

e-mail: [marcell.toth@med.uni-heidelberg.de](mailto:marcell.toth@med.uni-heidelberg.de)

**Supplementary Table 1. List of used antibodies**

| Primary Antibodies (clone) | Source/Company | Catalog number |
| --- | --- | --- |
| Amyloid A (mc1) | Agilent/Dako (Santa Clara, USA) | M0759 |
| Beta-Catenin (14) | Roche (Basel, Switzerland) | 760-4242 |
| Fatty-acid binding protein | Sigma-Aldrich (St. Louis, USA) | HPA028275 |
| Glutamine synthetase (GS-6) | Roche | 760-4898 |

**Supplementary Table 2. Patient and sample characteristics**

| Patient/  Sample | Age/Sex | Tumor type | Tumor cellularity  [%] |
| --- | --- | --- | --- |
| Patient 1  Sample 1 | 20/F | Moderately differentiated adenocarcinoma of the rectum | 70 |
| Patient 1  Sample 2 | 20/F | Multiple hepatocellular adenomas, NOS | 35 |
| Patient 1  Sample 3 | 20/F | Multiple hepatocellular adenomas, NOS | 80 |
| Patient 1  Sample 4 | 20/F | Multiple hepatocellular adenomas, NOS | 80 |
| Patient 1  Sample 5 | 20/F | Tumor free  lymph node | n.a. |
| Patient 2 | 22/F | Hepatocellular adenoma | 80 |
| Patient 3 | 57/M | Hepatocellular adenoma | 60 |

n.a.: not applicable

**Supplementary Table 3. Histomorphological characteristics of analyzed HCAs**

| **Sample** | **Architecture** | **Steatosis** | **Balloning** | **Inflammation** | **Reticulin loss** | **Myxoid changes** | **Sinusoidal dilation** | **Peliosis** | **Portal tracts-like structures** | **Unpaired arteries** | **Bile ducts** |
| --- | --- | --- | --- | --- | --- | --- | --- | --- | --- | --- | --- |
| P1/HCA1 | Trabecular | 3+ | - | 1+ | - | - | - | - | + | + | - |
| P1/HCA2 | Trabecular | 3+ | - | 1+ | - | - | - | - | + | + | - |
| P1/HCA3 | Trabecular | 3+ | - | 1+ | - | - | - | - | + | + | - |
| P2 | Trabecular | - | - | 3+ | - | - | 3+ | 3+ | + | + | - |
| P3 | Trabecular | - | - | 3+ | - | - | 2+ | 2+ | + | + | - |

P: patient; HCA: hepatocellular adenoma; +: feature is present; 1+: moderate; 2+: moderate; 3+: strong; -: feature is missing

**Supplementary Table 4. Overview of all mutations detected in the study cohort**

| Patient/  Sample | Gene | Exon | Mutation  (c.DNA) | Mutation  (protein) | Allele frequency [%] | Variant |
| --- | --- | --- | --- | --- | --- | --- |
| Patient 1  Sample 1  (Moderately  differentiated  adenocarcinoma  of the rectum) | APC  (NM_000038.6)  APC  (NM_000038.6)  JUN  (NM_002228.4)  MAP3K1  (NM_005921.2)  SMAD4  (NM_005359.5)  IDH1  (NM_005896.3)  PIK3CA  (NM_006218.4)  KRAS  (NM_004985.5)  ROS1  (NM_002944.2)  BIVM-ERCC5  (NM_001204425.1)  SMAD4  (NM_005359.5) | 7  16  1  Intr14  Intr9  4  10  2  17  22  12 | c.694C>T  c.3916_3917insGGTATT  c.501dup  c.3667-1G>A  c.1139+1G>A  c.394C>T  c.1624G>A  c.35G>A  c.2588G>A  c.4252C>T  c.1610A>T | p.Arg232*  p.Glu1306fs*3  p.Pro168fs*142  p.spl?  p.spl?  p.Arg132Cys  p.Glu542Lys  p.Gly12Asp  p.Arg863Gln  p.Arg1418Trp  p.Asp537Val | 19.2  35.2  7.9  23.7  23.7  13.7  28.9  22  25.1  58.7  25.7 | Lof  Lof  Lof  Lof predicted  Lof predicted  act  act  act  VUS  VUS  VUS |
| Patient 1  Sample 2  (Hepatocellular  adenoma) | APC  (NM_000038.6)  APC  (NM_000038.6)  BIVM-ERCC5  (NM_001204425.1) | 16  16  22 | c.4630G>T  c.3916_3917insGGTATT  c.4252C>T | p.Glu1544*  p.Glu1306fs*3  p.Arg1418Trp | 3.8  43.3  43 | Lof  Lof  VUS |
| Patient 1  Sample 3  (Hepatocellular  adenoma) | APC  (NM_000038.6)  APC  (NM_000038.6)  BIVM-ERCC5  (NM_001204425.1) | 16  16  22 | c.4666dupA  c.3916_3917insGGTATT  c.4252C>T | p.Thr1566fs*3  p.Glu1306fs*3  p.Arg1418Trp | 30  47.8  43.8 | Lof  Lof  VUS |
| Patient 1  Sample 4  (Hepatocellular  adenoma) | APC  (NM_000038.6)  APC  (NM_000038.6)  BIVM-ERCC5  (NM_001204425.1) | 16  16  22 | c.4729G>T  c.3916_3917insGGTATT  c.4252C>T | p.Glu1577*  p.Glu1306fs*3  p.Arg1418Trp | 16.3  44  45 | Lof  Lof  VUS |
| Patient 1  Sample 5  (Tumor free  lymph node) | APC  (NM_000038.6)  BIVM-ERCC5  (NM_001204425.1) | 16  22 | c.3916_3917insGGTATT  c.4252C>T | p.Glu1306fs*3  p.Arg1418Trp | 44  44.1 | Lof  VUS |
| Patient 2  (Hepatocellular  adenoma) | APC  (NM_000038.6)  APC  (NM_000038.6)  APC  (NM_000038.6)  PRKN  (NM_004562.3)  NFE2L2  (NM_001145413.3)  BCL2L11  (NM_001204109.1)  RICTOR  (NM_152756.5)  NOTCH1  (NM_017617.5)  TCF7L2  (NM_030756.5)  FANCF  (NM_022725.3)  STAT5A  (NM_003152.3)  BORCS8-MEF2B  (NM_005919.4)  NCOA  (NM_006534.4) | 16  16  16  7  2  3  28  17  4  1  7  8  20 | c.4544_4547dup  c.3467_3470del  c.5540C>T  c.766C>T  c.41T>G  c.406G>A  c.2728C>T  c.2636G>T  c.478A>G  c.940C>G  c.676C>G  c.634C>T  c.3775_3783del | p.Gln1517fs*17  p.Glu1156fs*8  p.Thr1847Met  p.Arg256Cys  p.Leu14Arg  p.Glu136Lys  p.Arg910Cys  p.Arg879Leu  p.Ile160Val  p.Leu314Val  p.Arg226Gly  p.Pro212Ser  p.Gln1270_Gln1272del | 11.6  41.4  40.2  44.7  16.1  34.5  45.1  50.1  47.9  50.5  46.1  47.2  28.2 | Lof  Lof  VUS  Lof predicted  Act  VUS  VUS  VUS  VUS  VUS  VUS  VUS  VUS |
| Patient 3  (Hepatocellular  adenoma) | APC  (NM_000038.6)  APC  (NM_000038.6)  PARP1  (NM_001618.4)  NSD1  (NM_022455.4)  MGA  (NM_001164273.1)  AXIN2  (NM_004655.4)  JAK3  (NM_000215.3)  KMT2B  (NM_014727.2) | 16  16  22  5  17  8  16  3 | c.4033G>T  c.4391_4392del  c.2938A>C  c.1825C>T  c.6784A>G  c.1985T>C  c.2164G>A  c.1802G>A | p.Glu1345*  p.Ser1465fs*3  p.Asn980His  p.Pro609Ser  p.Lys2262Glu  p.Leu662Pro  p.Val772Ile  p.Arg601Gln | 11  43.5  46.9  50.4  47.4  47.1  47.8  51.9 | Lof  Lof  VUS  VUS  VUS  VUS  VUS  VUS |

Lof: Loss of function; act: activating; VUS: variant of unknown significance; Intr: Intron
